# Clinical Mentorship in Nursing Practice in the UAE: A Mixed‐Methods Study of Mentor Perspectives on Effectiveness, Training, and Support

**DOI:** 10.1155/jonm/3224685

**Published:** 2026-06-23

**Authors:** Jacqueline Maria Dias, Semiyu Adejare Aderibigbe, Mini Sara Abraham

**Affiliations:** ^1^ Department of Nursing, College of Health Sciences, University of Sharjah, Sharjah, UAE, sharjah.ac.ae; ^2^ Department of Education, College of Arts, Humanities, and Social Sciences, University of Sharjah, Sharjah, UAE, sharjah.ac.ae

**Keywords:** benefits, challenges, clinical mentorship, compensation, mentors, nursing, students, workplace

## Abstract

**Background:**

Clinical mentorship is a cornerstone of nursing education, supporting the development of clinical competence, professional identity, and readiness for practice. Although mentorship has been widely studied internationally, evidence from Arab countries remains limited. This study examined nursing professionals’ perceptions of clinical mentorship and explored factors influencing mentorship effectiveness within healthcare settings in the United Arab Emirates (UAE).

**Methods:**

A sequential explanatory mixed‐methods design, grounded in a pragmatic worldview, was employed. Quantitative data were collected through a cross‐sectional survey of 75 nursing professionals working across healthcare facilities in the UAE. Descriptive and inferential statistics were used to examine mentorship experiences and perceived effectiveness. Qualitative data from open‐ended survey responses were analyzed thematically to explain and enrich quantitative findings. Integration occurred during interpretation through the comparison of quantitative and qualitative results.

**Results:**

Most participants (76.0%, *n* = 57) reported that mentorship positively influenced students’ preparedness for clinical practice under four themes. The themes are enhanced clinical competence, bridging theory and practice, the importance of supportive mentor behaviors, and the value of continuous learning support. Participants also identified challenges that affected mentorship effectiveness, including communication barriers, limited student engagement, time constraints, and inadequate mentor preparation. Among respondents who completed the evaluation stress item (*n* = 44), 41.3% (*n* = 31) reported no stress when evaluating students, whereas 17.3% (*n* = 13) reported evaluation‐related stress associated with unclear assessment criteria, workload pressures, and competing clinical responsibilities. More than half of participants (54.7%, *n* = 41) preferred a combination of compensation approaches, particularly continuing professional development opportunities and formal recognition.

**Conclusion:**

Clinical mentorship is an important contributor to nursing students’ preparedness and professional development. Strengthening mentor training, clarifying evaluation processes, addressing workload pressures, and implementing meaningful recognition strategies may enhance the effectiveness and sustainability of mentorship in clinical practice settings.

## 1. Introduction

In a recent report, the World Health Organization (WHO) indicated an increase in the global nursing workforce from 27.9 million in 2018 to 29.8 million in 2023. However, the organization still estimates a global shortage of approximately 5.8 million nurses, underscoring the persistent need for strategies that enhance workforce development, retention, and professional support systems [1]. Clinical mentorship is widely recognized as an effective strategy and has become a fundamental component of nursing education and workforce development. Clinical mentorship is defined as a developmental relationship in which an experienced practitioner provides guidance, feedback, support, and professional role modeling to facilitate the growth and competence of less experienced learners [2]. Through mentorship, nursing students are assisted in translating theoretical knowledge into clinical practice, developing professional confidence, strengthening critical thinking skills, and acquiring the competencies necessary for safe and effective patient care [3]. Effective mentorship further contributes to professional identity formation, smoother transitions into practice, and the development of a nursing workforce equipped to address increasingly complex healthcare demands [3–5].

Substantial evidence supports the value of clinical mentorship. Quantitative studies have identified positive associations between mentorship and improved clinical competence, enhanced clinical decision‐making, greater engagement with evidence‐based practice, increased job satisfaction, and stronger workforce retention [6–10]. Structured mentorship programs are also linked to improved patient outcomes and reduced staff turnover, indicating benefits that extend beyond individual learners to healthcare organizations and the broader healthcare system [9, 10]. However, quantitative research often emphasizes measurable outcomes and offers limited insight into the contextual and interpersonal processes through which mentorship influences learning and professional development.

Qualitative research offers a deeper understanding of mentorship experiences by examining the perspectives of both mentors and mentees. Previous studies emphasize the significance of supportive mentor–student relationships, effective communication, psychological safety, and professional role modeling in facilitating successful learning experiences [11, 12]. Nursing students often identify mentorship as central to developing confidence, professional identity, and a sense of belonging in clinical environments. Mentors, in turn, report professional satisfaction but also face challenges related to workload pressures, time constraints, and competing clinical responsibilities [11, 13, 14]. Qualitative findings further underscore the impact of cultural and linguistic diversity on mentoring relationships, highlighting the necessity for culturally responsive approaches that support learners from diverse backgrounds [15]. While qualitative studies provide valuable insights into lived experiences, their findings are frequently context‐specific and may not capture broader patterns across larger populations.

The United Arab Emirates (UAE) represents a particularly relevant context for the study of clinical mentorship. The healthcare workforce in the UAE is marked by significant cultural, linguistic, and educational diversity, with multinational teams providing care in a rapidly expanding healthcare sector [5]. In this setting, effective mentorship is essential for supporting nursing students’ transition into clinical practice, promoting professional integration, and fostering culturally competent care. Studies conducted in the UAE and the broader Gulf region indicate that mentorship positively influences students’ confidence, workplace adaptation, clinical competence, and professional development [3, 5, 16]. Nevertheless, there is limited evidence regarding mentors’ perspectives on mentorship effectiveness, the challenges they face, and the types of support needed to sustain mentoring roles in contemporary healthcare environments.

Although interest in clinical mentorship is increasing, relatively few studies have combined quantitative and qualitative approaches to achieve a comprehensive understanding of mentorship effectiveness. This gap is especially notable in the UAE, where the cultural and organizational features of the healthcare system may shape both the benefits and challenges of mentorship. A mixed‐methods approach, which integrates quantitative data with qualitative insights, offers a more complete understanding of mentorship experiences than either method alone. Accordingly, this study utilized a sequential explanatory mixed‐methods design to investigate nursing professionals’ perceptions of clinical mentorship in the UAE, focusing on mentorship effectiveness, challenges encountered by mentors, stress related to student evaluations, and preferred forms of mentor recognition and support.

The study addressed the following research questions:

1. How effective is mentorship in preparing nursing students for clinical practice in the UAE?
2. What challenges do clinical mentors encounter when mentoring nursing students?
3. What factors contribute to stress during student evaluation, and what strategies may reduce this stress?
4. What forms of compensation or recognition are preferred by clinical mentors?

Additionally, the quantitative component of the study tested the following null hypotheses:

1. H_01_: Participants’ educational level is not significantly associated with their perceptions of the benefits and challenges of clinical mentorship.
2. H_02_: Participants’ years of clinical experience are not significantly associated with their perceptions of the benefits and challenges of clinical mentorship.

## 2. Methods and Materials

### 2.1. Research Design

A sequential explanatory mixed‐methods design was employed, integrating quantitative survey data with qualitative insights from open‐ended survey responses [15]. The research was grounded in a pragmatic worldview, which posits that combining quantitative and qualitative approaches yields a more comprehensive understanding of complex phenomena than either approach alone [15, 17]. Given that clinical mentorship is shaped by individual, relational, organizational, and contextual factors, a mixed‐methods approach was deemed suitable for examining both measurable outcomes and mentors’ experiences.

The sequential explanatory design enabled initial identification of patterns related to mentorship effectiveness, challenges, evaluation‐related stress, and compensation preferences through quantitative analysis, followed by the use of qualitative findings to explain and contextualize these results [15, 18]. Although quantitative and qualitative data were collected concurrently within a single survey instrument, analysis proceeded sequentially: Quantitative findings were analyzed first, followed by inductive thematic analysis of open‐ended responses. Thus, the sequential aspect of the design pertains to the order of analysis and interpretation, rather than to temporally distinct phases of data collection [19].

The quantitative component utilized a descriptive cross‐sectional survey design appropriate for examining participants’ perceptions and experiences at a single point in time [20]. The qualitative component comprised open‐ended survey responses analyzed using inductive thematic analysis [21]. Integration was achieved during interpretation through narrative weaving and the development of joint displays, allowing qualitative findings to explain and expand upon quantitative patterns [22–24]. This approach generated meta‐inferences that contributed to a more comprehensive understanding of mentorship experiences within the UAE nursing context.

### 2.2. Setting and Participants

The study was conducted across five healthcare facilities in the UAE, comprising two tertiary hospitals, two specialist centers, and one community health facility. These sites were selected to reflect the diversity of clinical environments in which nursing mentorship occurs.

Eligible participants were registered nurses with a minimum of 2 years of clinical experience who had directly supervised undergraduate nursing students during clinical placements within the previous 12 months. Nurses without mentoring responsibilities or those not involved in student supervision during the study period were excluded.

Participant recruitment utilized both convenience and purposive sampling strategies [25, 26]. Convenience sampling enabled access to eligible nursing staff across participating facilities, while purposive sampling ensured inclusion of participants with direct experience mentoring nursing students. Questionnaire distribution across participating sites was facilitated by a member of the research team (MSA), who served as the clinical practicum coordinator.

A total of 90 questionnaires were distributed electronically via institutional email. Seventy‐five completed questionnaires were returned, yielding a response rate of 83.3%. Because the study sought preliminary evidence on mentorship experiences among eligible mentors across participating institutions, all available mentors who met the inclusion criteria during the data collection period were invited to participate. The sample size was therefore determined pragmatically based on the available population and is consistent with exploratory mixed‐methods research conducted in healthcare settings [20].

### 2.3. Instrument Development and Data Collection Tool

Data collection was conducted using a structured, self‐administered questionnaire developed specifically for this study and administered in English, the primary language of professional communication within UAE healthcare institutions.

Questionnaire development adhered to recommendations outlined by Artino et al. [27]. Instrument development proceeded in four stages: (1) review of mentorship and nursing education literature; (2) generation of items aligned with study objectives and research questions; (3) expert review for content relevance, clarity, and comprehensiveness; and (4) pilot testing with practicing nurse mentors.

The final instrument consisted of two sections.

Section 1 collected demographic and professional information, including educational level, years of clinical experience, mentorship experience, and previous mentorship training.

Section 2 examined mentorship effectiveness, perceived benefits, challenges, evaluation‐related stress, and preferred forms of mentor recognition and support using a combination of dichotomous, multiple‐choice, Likert‐scale, and open‐ended questions (Table 1).

**TABLE 1:** Core survey items by response format.

No.	Survey item	Response format
1	Have you received formal mentorship training?	Dichotomous (yes/no)
2	If yes, what was the duration of your training?	Multiple choice: 1 day; 2–4 days; 1 week; more than 1 week
3	How effective do you believe mentorship is in preparing students for clinical practice?	5‐point Likert scale (1 = *strongly disagree* to 5 = *strongly agree*)
4	What challenges have you experienced in your role as a mentor?	5‐point Likert scale + open‐ended
5	What forms of compensation or support would enhance your willingness and capacity to mentor?	Open‐ended

### 2.4. Content Validity, Pilot Testing, and Reliability

Content validity was established through a panel review by nursing academics and experienced clinical mentors. Panel members evaluated each item for relevance, clarity, comprehensiveness, and alignment with the study objectives. Recommendations resulted in minor revisions to wording and item sequencing.

The questionnaire was pilot tested with 10 nursing professionals who met the study inclusion criteria but were not included in the final sample. Pilot participants assessed item clarity, response burden, and overall usability. Minor modifications were implemented prior to commencement of the main study [27].

Internal consistency reliability was assessed using Cronbach’s alpha, yielding an overall coefficient of 0.54. Although this value is below the commonly cited threshold of 0.70, it is interpreted with caution because the instrument was intentionally designed to assess multiple conceptually distinct domains within a single instrument, including mentorship effectiveness, benefits, challenges, evaluation‐related stress, and compensation preferences [28]. George and Mallery suggest that alpha values between 0.50 and 0.60 may be acceptable in exploratory studies [29]. Item review during piloting did not identify problematic items; however, formal item‐total correlation analyses were not conducted, and this limitation is acknowledged. Findings from the quantitative component were further contextualized with qualitative data, providing methodological triangulation within the mixed‐methods design.

### 2.5. Trustworthiness and Rigor

Trustworthiness of the qualitative component was established using the criteria of credibility, dependability, confirmability, and transferability proposed by Lincoln and Guba [30].

Credibility was enhanced through repeated review of participants’ verbatim responses, independent coding by two researchers (Jacqueline Maria Dias and Semiyu Adejare Aderibigbe), and consensus discussions regarding coding decisions. Dependability was supported by maintaining an audit trail documenting coding decisions, theme development, and analytic discussions. Confirmability was strengthened through reflexive discussions among the research team to minimize the influence of personal assumptions on interpretation. Transferability was supported by providing detailed descriptions of the study context, participant characteristics, and mentorship environment.

Additionally, rigor within the mixed‐methods component was supported through the integration of findings, the development of meta‐inferences, and the use of qualitative data to explain quantitative results, consistent with contemporary recommendations for mixed‐methods health research [22–24, 31]. Following these procedures lends credibility and dependability to the study.

### 2.6. Data Collection Procedure

Data collection occurred over a 3‐month period following institutional ethical approval (REC24‐01‐02‐01‐S). Eligible participants received an electronic invitation containing study information and a link to the questionnaire. Completion of the questionnaire was considered to indicate informed consent. Reminder emails were distributed periodically to maximize participation. Besides, all responses were anonymized prior to analysis and stored on password‐protected systems accessible exclusively to the research team.

### 2.7. Data Analysis

Quantitative data were analyzed using IBM SPSS Statistics Version 26. Descriptive statistics, including frequencies, percentages, means, and standard deviations, were used to summarize participant characteristics and survey responses.

Prior to inferential analyses, assumptions relevant to each statistical test were assessed. Normality was evaluated using the Shapiro–Wilk test. Expected cell frequencies were examined before chi‐square analyses, and nonparametric procedures were applied when assumptions for parametric testing were not met. Pearson and Spearman correlation analyses, chi‐square tests, and analysis of variance were conducted as appropriate to address the study objectives. Effective sample sizes for individual analyses are reported in the Results section due to item‐level missing data.

Qualitative data were analyzed independently by two researchers (Jacqueline Maria Dias and Semiyu Adejare Aderibigbe) using Braun and Clarke’s six‐phase thematic analysis framework [21]. The analysis process included familiarization with the data, generation of initial codes, identification of candidate themes, review of themes, definition and naming of themes, and preparation of the final report. Coding was conducted independently, followed by consensus meetings to resolve discrepancies and refine thematic interpretations. As qualitative data were derived from brief open‐ended survey responses rather than interviews or focus groups, data saturation was not considered an explicit methodological objective. Instead, qualitative findings were used to provide contextual explanation and enrich quantitative results within the broader mixed‐methods design.

## 3. Results

### 3.1. Participant Characteristics

A total of 75 nursing professionals participated in the study. As shown in Table 2, most participants held a Bachelor of Science in Nursing qualification (*n* = 59, 78.7%). Participants were generally experienced clinicians, with the largest proportion reporting 11–15 years of clinical experience (*n* = 26, 34.7%). More than half of the sample were actively involved in mentoring nursing students (*n* = 42, 56.0%) and newly employed nursing staff (*n* = 47, 62.2%).

**TABLE 2:** Demographic characteristics of participants (*n* = 75).

Characteristic	*N*	%
*Educational level*		

BSN	59	78.7%
Diploma	9	12.0%
MSN	5	6.7%
PhD	1	1.3%
Missing	1	1.3%

*Years of clinical experience*		

1–5 years	7	9.3%
6–10 years	11	14.7%
11–15 years	26	34.7%
16–20 years	14	18.7%
21–25 years	6	8.0%
26–30 years	2	2.7%
30 years and above	1	1.3%
Missing	8	10.7%

*Mentorship role*	*Yes n (%)*	*No n (%)*

Mentor for new staff	47 (62.2%)	28 (37.8%)
Mentor for nursing students	42 (56.0%)	33 (44.0%)

*Note:* Mentorship role percentages are of the total sample (*n* = 75).

Abbreviations: BSN, Bachelor of Science in Nursing; MSN, Master of Science in Nursing.

The demographic profile demonstrates that participants had considerable clinical and mentoring experience, which contributed to an informed perspective on mentorship practices in UAE healthcare settings.

### 3.2. Research Question 1: How Effective is Mentorship in Preparing Nursing Students for Clinical Practice in the UAE?

Participants were asked about mentorship training, training duration, and their perceptions of mentorship effectiveness. Table 3 presents the outcome of the analyzed responses of the mentors.

**TABLE 3:** Mentorship training and duration.

Training received	*n*	%	1 day	> 1 week	2–4 days	1 week	Missing
Yes	46	61.3	45	14	8	2	—
No	28	37.3	—	—	—	—	—
Missing	1	1.3	—	—	—	—	6
Total	75	100	—	—	—	—	—

*Note:* Duration columns are not mutually exclusive; participants who received training across multiple formats are counted in each applicable column. The missing column (*n* = 6) applies to training duration data only, among those who confirmed receiving training.

Of the 75 participants, 61.3% (*n* = 46) reported receiving formal mentorship training. Even so, training duration varied considerably, with most participants indicating relatively brief training experiences. These results indicate variation in mentor preparation across participating institutions.

Qualitative responses describing the perceived benefits of mentorship generated four themes: professional growth, knowledge sharing, support and guidance, and confidence building (Table 4).

**TABLE 4:** Perceived benefits of mentorship to nursing students.

Theme	Key benefits	Representative participant comments
Professional growth	Career advancement; professional development	*“To be able to share knowledge, skills, attitude, and experience enhances professional growth.” (Mentor 3)* *“It helps in understanding deeper professional roles and future career possibilities.” (Mentor 3)*

Knowledge sharing	Exchange of knowledge; continuous learning	*“Sharing of experience and knowledge which enriches both mentor and mentee.” (Mentor 22)* *“Mentorship provides a platform for continuous professional education and sharing of up-to-date practices.” (Mentor 15)*

Support and guidance	Guidance in clinical settings; emotional support	*“Guidance through complex clinical situations and career decisions.” (Mentor 40)* *“Support in times of professional uncertainty and personal growth.” (Mentor 34)*

Confidence building	Increased self‐assurance; empowerment	*“Helps build confidence in newer or less experienced staff.” (Mentor 52)* *“Mentorship empowers nurses by building their confidence to take on more responsibilities.” (Mentor 43)*

The findings confirm that clinical mentorship contributes to student development across professional, cognitive, relational, and affective domains, reflecting its multidimensional role in nursing education.

When asked whether mentorship effectively prepares nursing students for clinical practice, 76.0% (*n* = 57) responded affirmatively. Table 5 shows the results, illustrating the two perspectives.

**TABLE 5:** Effectiveness of clinical training of nursing students under mentorship.

Response type	Detail	*n*	Themes and representative comments
Affirmative responses	Mentorship enhances preparedness	57	Enhanced competence: *“Yes, with practical knowledge and observation, they will be more competent.” (Mentor 12)* Bridging theory and practice: *“Practical, real-world experience with mentorship enhances skills, decision-making, and confidence.” (Mentor 18)* Positive mentor approach: *“Yes, a good mentor can really bring out the best in a student.” (Mentor 43)* Support for ongoing learning: *“Ongoing mentorship helps maintain a high standard of care and fosters continuous improvement.” (Mentor 65)*

Nonaffirmative responses	Uncertain or disagreement	18	Participants did not provide elaborated qualitative responses; quantitative frequency recorded only.

Total responses	All participants	75	

Clinical training supported by mentorship significantly enhances the competence of nursing students. Mentorship helps bridge the gap between theoretical knowledge and practical application, improving students’ skills, decision‐making abilities, and confidence. This structured support system empowers students and plays a pivotal role in developing a more competent and resilient nursing workforce. As shown in Table 5, participants consistently described mentorship as facilitating the application of theoretical knowledge in clinical environments and enhancing both confidence and professional decision‐making. The findings indicate that mentorship is widely regarded as an effective strategy for preparing nursing students for clinical practice. Quantitative data demonstrate high levels of perceived effectiveness, while qualitative data provide insight into the ways mentorship supports competence development and professional growth.

### 3.3. Research Question 2: What Challenges Do Clinical Mentors Encounter When Mentoring Nursing Students?

Participants identified several challenges associated with the mentoring role. However, as shown in Table 6, four themes emerged.

**TABLE 6:** Challenges faced by mentors of nursing students in clinical training.

Theme	*n*	%	Representative comment 1	Representative comment 2
Time constraints and workload	25	33.3	*“Busy working environment.” (Mentor 5)*	*“During working time will be less effective.” (Mentor 38)*
Communication barriers	21	28.0	*“Communication barriers.” (Mentor 18)*	*“Communication barrier.” (Mentor 24)*
Lack of student engagement	18	24.0	*“Interest of the student in learning, less interest, more challenges.” (Mentor 35)*	*“Lack of focus on following the mentor seriously.” (Mentor 37)*
Insufficient training and unclear expectations	11	15.0	*“Without proper training and discussions, knowing what students need to focus on would be a challenge.” (Mentor 54)*	*“Understand the limits and boundaries.” (Mentor 66)*
Total	75	100		

From the table, time constraints and workload represented the most frequently reported challenge (33.3%, *n* = 25), followed by communication barriers (28.0%, *n* = 21). Participants often described the difficulty in balancing mentorship responsibilities with demanding clinical workloads. Thus, mentorship challenges were primarily organizational rather than individual. Workload pressures, communication difficulties, and insufficient preparation consistently emerged as barriers to effective mentoring.

### 3.4. Research Question 3: What Factors Contribute to Stress During Student Evaluation, and What Strategies May Reduce This Stress?

Participants were asked whether evaluating nursing students was stressful and to propose strategies that might reduce evaluation‐related stress. Table 7 illustrates mentors’ perspectives on the question.

**TABLE 7:** Stressfulness of student evaluation: mentor responses.

Response	*n*	% of sample	Representative comments
Not stressful	31	41.3	*“No.” (Mentor 70) “None.” (Mentor 60) “Not at all.” (Mentor 42)*

Stressful	13	17.3	*“During busy shifts will be so stressful.” (Mentor 16)* *“Lack of clear evaluation criteria adds to the stress.” (Mentor 47)*

Did not respond	31	41.3	Item was optional; nonresponse may reflect discomfort disclosing stress or perceived non‐applicability.

Total	75	100	

As Table 7 revealed, among participants who responded to the evaluation stress item (*n* = 44), 31 (70.5%) indicated that evaluation was not stressful, while 13 (29.5%) reported experiencing evaluation‐related stress. Relative to the total sample, these figures correspond to 41.3% and 17.3%, respectively. Thirty‐one participants did not respond to this item. Because the question was optional, nonresponses were retained and reported for transparency.

Furthermore, qualitative analysis identified five strategies for reducing evaluation‐related stress. Table 8 shows the themes and supporting comments.

**TABLE 8:** Mentor‐proposed strategies to reduce evaluation‐related stress.

Strategy	Representative participant comments
Student self‐evaluation and re‐evaluation	*“Self-evaluation by the student then re-evaluation by mentor.” (Mentor 17)* *“Encouraging students to assess their own performance helps in reducing mentor’s stress.” (Mentor 11)*

Open communication	*“Open communication.” (Mentor 29)* *“Maintaining open lines of communication eases the evaluation process.” (Mentor 28)*

Time management and organization	*“Be organized and proper time management helps to reduce stress.” (Mentor 16)* *“Proper scheduling and organization can alleviate stress.” (Mentor 45)*

Building trust	*“Building trust.” (Mentor 48)* *“Creating a trusting environment reduces stress for both mentors and students.” (Mentor 33)*

Workload adjustment and peer support	*“Group sharing and providing break time.” (Mentor 14)* *“Balancing workload and mentoring duties is crucial to reduce stress.” (Mentor 64)*

As shown in Table 8, a minority of respondents reported evaluation‐related stress, most commonly linked to workload pressures and unclear evaluation expectations. Participants proposed both relational and organizational strategies to support mentors during student assessment.

### 3.5. Research Question 4: What Forms of Compensation or Recognition Are Preferred by Clinical Mentors?

Participants identified their preferred forms of recognition and compensation for mentoring activities. Table 9 presents the results.

**TABLE 9:** Preferred methods of compensation for clinical mentors (*n* = 75).

Compensation method	*n*	%	Representative comment
Combination of all methods	41	54.7	*“A mix of various compensation methods ensures that mentors feel valued and adequately rewarded.” (Mentor 27)*
Continuing education opportunities	25	33.3	*“Providing opportunities for continuing education helps mentors stay updated and motivated.” (Mentor 5)*
Formal recognition in annual appraisals	4	5.3	*“Including mentoring in the annual appraisal recognises the efforts and contributions of mentors.” (Mentor 16)*
Other methods	3	4.0	*“Other unspecified methods were also suggested, indicating a diverse range of preferences.” (Mentor 32)*
No response	2	2.7	Item was optional; nonresponses not included in chi‐square analysis.
Total	75	100	

More than half of participants (54.7%, *n* = 41) preferred a combination of compensation approaches. Continuing education opportunities were the second most frequent preferred option (33.3%, *n* = 25). Participants emphasized that effective recognition should encompass both professional development opportunities and formal acknowledgment of mentoring contributions. The findings, therefore, indicate that mentors prefer flexible, multifaceted recognition strategies instead of single forms of compensation.

### 3.6. Hypothesis Testing

H_01_: Participants’ educational level is not significantly associated with their perceptions of the benefits and challenges of clinical mentorship.

Spearman correlation analyses were conducted to examine the relationship between educational level and perceived mentorship benefits and challenges, with the findings presented in Table 10.

**TABLE 10:** Association between demographic factors and perceived benefits and challenges of mentorship (*n* = 75).

Demographic factor	Benefits score (*r*)	Challenges score (*r*)	*p* value (benefits)	*p* value (challenges)
Education level	−0.036	−0.016	0.770	0.898
Years of experience	−0.140	−0.098	0.243	0.410

No statistically significant associations were identified between educational level and perceived benefits (*p* > 0.05) or challenges (*p* > 0.05). Therefore, H_01_ was retained.

H_02_: Participants’ years of clinical experience are not significantly associated with their perceptions of the benefits and challenges of clinical mentorship.

Spearman correlation analyses were conducted to examine associations between years of clinical experience and perceived mentorship benefits and challenges. As shown in Table 11, no statistically significant associations were identified between years of clinical experience and perceived benefits (*p* > 0.05) or challenges (*p* > 0.05). Therefore, H_02_ was retained.

**TABLE 11:** Effect of mentor training duration on perceived benefits and challenges of mentorship (ANOVA).

Dependent variable	Sum of squares	*df*	*F*‐statistic	*p* value
Benefits score	14561.87	1	2.28	0.136
Challenges score	889.92	1	0.29	0.590

### 3.7. Integration of Data Sets and Meta‐Inference

To integrate the quantitative and qualitative strands, a joint display was developed and presented in Table 12 to compare findings across the four research questions and to generate meta‐inferences derived from the systematic integration of both data strands [15, 19, 22–24].

**TABLE 12:** Integration of quantitative and qualitative findings by research questions.

Research question	Quantitative strand (survey, *n* = 75)	Qualitative strand (open‐ended responses)	Meta‐inference (integrated interpretation)
RQ1: How effective is mentorship in preparing nursing students for clinical practice?	76% (*n* = 57) affirmed effectiveness. 61.3% (*n* = 46) received training; most limited to 1 day. Pearson’s *r* = 0.68, *p* < 0.01 between training and perceived effectiveness.	Themes: enhanced competence; theory–practice bridge; positive mentor approach; ongoing learning support. *“Yes, with practical knowledge and observation, they will be more competent.” (Mentor 12)*	Quantitative findings indicate a high level of perceived mentorship effectiveness. Qualitative findings explain this effectiveness through enhanced competence, integration of theory and practice, supportive mentor–mentee relationships, and continuous learning. Together, the findings suggest that mentorship plays an important role in preparing nursing students for clinical practice and that mentor training may strengthen these positive outcomes.

RQ2: 2. What challenges do clinical mentors encounter when mentoring nursing students?	Chi‐square (*χ* ^2^ = 12.76, *p* < 0.05): time constraints 33.3% (*n* = 25); communication 28.0% (*n* = 21); engagement 24.0% (*n* = 18); training 15.0% (*n* = 11).	Themes: Communication barriers; lack of student engagement; time constraints; unclear expectations. *“Busy working environment.” (Mentor 5)*	Both strands converge in identifying workload, time constraints, communication barriers, and student engagement issues as major challenges. These findings suggest that mentorship challenges are influenced by organizational and educational factors that may require institutional support and targeted interventions.

RQ3: 3. What factors contribute to stress during student evaluation, and what strategies may reduce this stress?	17.3% (*n* = 13) reported evaluation stress; 41.3% (*n* = 31) reported none; 31 did not respond. Spearman’s *ρ* = 0.52, *p* < 0.01: workload intensity correlated with stress.	Themes: Student self‐evaluation and re‐evaluation; open communication; time management and organization; building trust; workload adjustment and peer support. *“Self-evaluation by the student then re-evaluation by mentor.” (Mentor 17)*	Quantitative findings indicate that mentorship‐related stress is experienced by some mentors and is associated with workload intensity. Qualitative findings identify practical strategies to reduce stress, including communication, workload management, trust‐building, and student self‐evaluation. Together, the findings suggest that mentorship stress can be mitigated through both relational and organizational support mechanisms.

RQ4: What forms of compensation or recognition are preferred by clinical mentors?	54.7% (*n* = 41) preferred combination model. 33.3% (*n* = 25) continuing education; 5.3% (*n* = 4) formal recognition. Chi‐square (*χ* ^2^ = 9.82, *p* < 0.05) confirmed preference for mixed model.	Themes: Professional development; formal recognition; mixed incentives. *“A mix of various compensation methods ensures that mentors feel valued.” (Mentor 27)*	Quantitative and qualitative findings converged in demonstrating a preference for multifaceted compensation approaches. Mentors valued a combination of professional development opportunities, formal recognition, and other incentives. These findings suggest that flexible, multicomponent compensation frameworks may be more effective than reliance on a single reward mechanism.

*Note:* Meta‐inferences were developed through the integration of quantitative and qualitative findings using a convergent mixed‐methods approach. Qualitative quotations are representative examples of broader themes identified during thematic analysis. Statistical significance was assessed at *p* < 0.05.

The joint display demonstrates substantial convergence between the quantitative and qualitative findings. Quantitative results identified broad patterns related to mentorship effectiveness, challenges, evaluation stress, and compensation preferences, while qualitative findings offered contextual explanations for these patterns. Across both strands, mentorship was perceived as beneficial for student preparedness. Organizational factors emerged as the primary barriers to effective mentoring, and participants emphasized the need for institutional support through training, workload management, and meaningful recognition mechanisms.

The integration of both data strands indicates that mentorship is valued as a mechanism for strengthening nursing students’ preparedness and professional development, highlighting its effectiveness. However, this depends largely on organizational conditions that support mentors, including adequate preparation, manageable workloads, clear evaluation processes, and formal recognition of mentoring contributions.

## 4. Discussion

### 4.1. Mentorship as a Mechanism for Clinical Readiness and Professional Development

The findings demonstrate that mentorship is broadly regarded as an effective strategy for preparing nursing students for clinical practice, with over three‐quarters of participants reporting enhanced student preparedness. Qualitative data further indicate that mentorship fosters professional growth, facilitates knowledge sharing, builds confidence, and supports the development of constructive learning relationships. Collectively, these results suggest that mentorship contributes to both the acquisition of clinical skills and the broader process of professional socialization.

The integrated findings align with Vygotsky’s concept of the Zone of Proximal Development, which emphasizes the role of knowledgeable guides in supporting learners as they progress beyond their current level of competence [32]. Within the clinical environment, mentors act as facilitators who scaffold students’ learning through guided participation, constructive feedback, and gradual exposure to increasingly complex clinical situations. Participants consistently described mentorship as helping students bridge the gap between classroom learning and clinical practice, a finding that also reflects principles of experiential learning theory [33]. Through observation, reflection, application, and feedback, students can transform theoretical knowledge into clinical competence.

The findings further support Bandura’s social learning theory and the concept of self‐efficacy [34, 35]. Participants described how encouragement, role modeling, and guided practice contributed to students’ confidence and readiness for professional practice. Similarly, the Communities of Practice framework suggests that professional learning occurs through participation within authentic professional environments [36, 37]. Mentorship, therefore, serves as an important mechanism through which students develop not only clinical competence but also professional identity and a sense of belonging within the nursing profession.

An important finding was the strong association between mentorship training and perceived mentorship effectiveness. Although most participants had received some form of mentor preparation, training was frequently limited to short‐duration programs. This suggests that mentorship training alone may be insufficient and that greater attention should be given to the quality, depth, and practical application of mentor development programs. Similar observations have been reported in studies demonstrating that structured mentor preparation improves mentoring confidence, educational competence, and mentoring effectiveness [6, 11, 38, 39].

The hypothesis testing results further strengthen these findings. Neither educational level nor years of clinical experience were significantly associated with perceived mentorship benefits or challenges. This suggests that effective mentorship may be less dependent on demographic characteristics and more strongly influenced by organizational support, mentor preparation, and the quality of mentor–student interactions. Consequently, mentorship development initiatives should be designed for mentors across all educational and experience levels rather than targeting specific demographic groups.

### 4.2. Organizational Barriers to Effective Mentorship

Despite the recognized benefits of mentorship, participants identified several challenges that may limit its effectiveness. The most frequently reported barriers included workload pressures, time constraints, communication difficulties, lack of student engagement, and insufficient mentor preparation. Quantitative findings identified workload and time limitations as the most commonly reported challenges, while qualitative findings provided insight into how these challenges affect day‐to‐day mentoring activities.

The predominance of workload‐related concerns suggests that barriers to effective mentorship are primarily organizational rather than individual. Participants frequently described difficulties balancing mentoring responsibilities with demanding clinical workloads. Similar findings have been reported internationally, where increasing patient acuity, staffing shortages, and competing clinical responsibilities have reduced opportunities for meaningful mentor–student interactions [11, 13, 39]. These findings indicate that mentorship cannot be viewed solely as an individual responsibility but requires organizational structures that provide protected time and adequate resources for mentoring activities.

Communication barriers emerged as another important challenge. Given the multicultural composition of the UAE healthcare workforce, communication difficulties may reflect differences in language, cultural expectations, educational backgrounds, and communication styles [8]. Previous research has similarly highlighted the importance of cultural competence and communication skills in supporting effective mentorship relationships within diverse clinical environments [5, 8]. Targeted mentor preparation programs that address intercultural communication may therefore improve mentoring effectiveness.

Lack of student engagement was also identified as a recurring concern. From an adult learning perspective, meaningful engagement occurs when learners perceive relevance, autonomy, and ownership of their learning experiences [40, 41]. Mentorship approaches that encourage shared goal setting, reflective learning, and active participation may therefore strengthen student engagement and maximize learning outcomes.

Finally, some participants reported uncertainty regarding mentorship expectations and evaluation responsibilities. This finding highlights the importance of formal mentorship frameworks that clearly define mentor roles, expectations, responsibilities, and assessment processes [6, 11]. Establishing standardized mentorship guidelines may reduce role ambiguity and enhance consistency in mentoring practices across healthcare settings.

### 4.3. Evaluation Stress and Support Strategies

The findings indicate that although many mentors did not perceive student evaluation as stressful, a significant proportion did report stress related to evaluation. Qualitative data suggest that this stress is frequently linked to workload pressures, competing responsibilities, and uncertainty about assessment expectations. The observed positive relationship between workload intensity and evaluation‐related stress further highlights the impact of organizational factors on mentors’ experiences.

The considerable number of nonresponses to the evaluation stress question may also be informative. Some mentors may have regarded the question as sensitive or may have been hesitant to disclose workplace‐related stress. While caution is necessary when interpreting this result, it underscores the importance of prioritizing mentor wellbeing and psychological safety within clinical learning environments.

Participants suggested several practical strategies to reduce evaluation‐related stress, such as student self‐assessment, open communication, workload management, trust‐building, and structured evaluation methods. These recommendations are consistent with previous studies that advocate for transparent assessment processes, standardized evaluation criteria, and mentor training in feedback and assessment practices [8, 14]. Incorporating student self‐evaluation alongside mentor assessment may further encourage reflective learning and decrease the perceived burden of assessment responsibilities.

These findings indicate that evaluation‐related stress is not an inherent aspect of mentorship but is shaped by modifiable organizational and procedural factors. Enhancing assessment clarity, alleviating workload pressures, and improving mentor preparation may therefore lead to more effective and less stressful evaluation processes.

### 4.4. Recognition, Motivation, and Sustainability of Mentorship

Recognition and compensation were identified as important factors for sustaining mentor engagement. Most participants expressed a preference for a combination of compensation approaches rather than reliance on a single incentive. Continuing professional development opportunities and formal recognition were regarded as particularly valuable forms of support.

These findings indicate that mentors perceive recognition as extending beyond financial rewards. Opportunities for professional growth, acknowledgment of mentoring contributions, and the inclusion of mentorship activities in performance appraisal processes were viewed as meaningful indicators that organizations value mentoring efforts. Previous mentorship research has similarly found that recognition, career development opportunities, and professional acknowledgment are associated with increased mentor satisfaction and retention [10, 11, 38, 39].

The preference for multifaceted recognition strategies reflects the diverse motivations influencing mentorship participation. Some mentors may prioritize career advancement, while others value professional recognition, continuing education, or workload adjustments. As a result, organizations may benefit from adopting flexible recognition frameworks that address varying mentor needs and preferences.

Given persistent nursing workforce challenges both globally and regionally [1], supporting and retaining skilled mentors is increasingly critical. Effective recognition strategies may enhance mentor satisfaction and contribute to the long‐term sustainability and quality of clinical mentorship programs.

## 5. Strengths, Limitations, and Future Directions

Several strengths are evident in this study. For instance, the sequential explanatory mixed‐methods design facilitated the integration of quantitative and qualitative data, yielding a more comprehensive understanding of mentorship experiences than either approach alone. Employing a joint display and developing meta‐inferences further strengthened the integration of findings and improved the interpretation of mentorship effectiveness, challenges, and support needs. Additionally, the study addressed a significant gap in the literature by examining mentorship from the perspective of clinical mentors in the UAE, a context marked by cultural, linguistic, and educational diversity. Participants were recruited from a range of healthcare settings, including tertiary hospitals, specialist centers, and community services, thereby increasing the relevance of the findings across various clinical environments. The 83.3% response rate indicates strong participant engagement and supports the representativeness of the findings among nurses actively involved in mentorship.

Despite the value of the study’s findings, several limitations should be considered when interpreting these findings. The overall Cronbach’s alpha coefficient of 0.54 indicates moderate internal consistency and falls below the commonly accepted threshold for established scales [28]. Although the instrument was designed to assess multiple dimensions of mentorship and was supported by qualitative findings within the mixed‐methods design, measurement error may have affected some quantitative estimates. Future research should refine the instrument using item‐total correlation analysis, construct validation, and test–retest reliability assessment. The use of convenience and purposive sampling limits the generalizability of the findings beyond the participating institutions. Although purposive sampling ensured that participants had relevant mentorship experience, the sample may not fully represent all nurse mentors within the UAE healthcare system.

A substantial proportion of participants did not respond to the item assessing evaluation‐related stress. This nonresponse may indicate discomfort discussing work‐related stress, concerns about professional perceptions, or a belief that the question was not applicable. Social desirability bias may have influenced responses to sensitive items [42]. The qualitative component relied on brief open‐ended survey responses rather than in‐depth interviews or focus groups. While these responses provided valuable contextual insights and supported the interpretation of quantitative findings, they did not permit the depth of exploration typically associated with standalone qualitative studies. Therefore, the qualitative findings should be interpreted as complementary explanatory data within the broader mixed‐methods framework.

The cross‐sectional design of this study limits the ability to draw causal conclusions about the relationships among mentorship training, mentorship experiences, and perceived effectiveness. Longitudinal research is required to examine how mentorship experiences and outcomes change over time and to evaluate the sustained impact of structured mentor development programs. Future studies should assess the effectiveness of comprehensive mentor training interventions using pre–post or quasi‐experimental designs to determine their influence on mentor competence, student outcomes, and mentor retention within clinical settings. Given the multicultural composition of the UAE healthcare workforce, future research should also investigate how cultural competence, language diversity, and intercultural communication affect mentoring relationships and student learning outcomes. Such research would support the development of culturally responsive and contextually relevant mentorship frameworks for nursing education and practice.

## 6. Conclusion

This mixed‐methods study provides evidence that clinical mentorship plays a pivotal role in preparing nursing students for professional practice within the UAE healthcare context. The integration of quantitative and qualitative findings demonstrated that mentorship contributes to the development of clinical competence, confidence, professional identity, and readiness for practice. Mentors consistently described mentorship as an important mechanism for supporting students’ transition from classroom learning to real‐world clinical environments, while quantitative findings confirmed widespread perceptions of mentorship effectiveness.

However, the study also identified several factors that limit the effectiveness and sustainability of mentorship programs. Workload pressures, time constraints, communication challenges, unclear expectations, and evaluation‐related stress were identified as key barriers that affect mentors’ ability to fully engage in their roles. The findings indicate that mentorship effectiveness is determined more by organizational conditions that support mentoring practice than by mentors’ educational level or years of experience.

The integrated findings underscore the necessity for healthcare institutions and nursing education programs to invest in structured mentor preparation, protected mentorship time, transparent evaluation processes, and meaningful recognition systems. The preference for flexible, multicomponent recognition strategies highlights the importance of acknowledging mentorship as a professional responsibility that requires institutional support, rather than relying solely on individual goodwill.

Besides, this study provides context‐specific evidence from the UAE, a healthcare environment characterized by cultural, linguistic, and educational diversity. By integrating quantitative breadth with qualitative depth, the study offers a comprehensive understanding of mentorship within this unique context and supplies evidence to inform mentorship policy, workforce development initiatives, and nursing education reforms. Strengthening mentorship practices can potentially enhance student learning, support mentor engagement, and contribute to the development of a competent and resilient nursing workforce capable of meeting evolving healthcare needs.

## Funding

This work was supported by University of Sharjah, 10.13039/100016714, 2201050381.

## Ethics Statement

The study received ethical approval from the University of Sharjah Ethical Review, Reference number: REC‐24‐01‐02‐01‐S. The study was carried out in accordance with institutional and international research ethics guidelines.

## Conflicts of Interest

The authors declare no conflicts of interest.

## Data Availability

The data that support the findings of this study are not publicly available due to the sensitive nature of the content, which may lead to the identification of individual mentors and clinical sites. To protect participant confidentiality and institutional privacy, access to the data is restricted.
